# Biomimetic Remineralization of an Extracellular Matrix Collagen Membrane for Bone Regeneration

**DOI:** 10.3390/polym14163274

**Published:** 2022-08-11

**Authors:** Raquel Osorio, Samara Asady, Manuel Toledano-Osorio, Manuel Toledano, Juan M. Bueno, Rosa M. Martínez-Ojeda, Estrella Osorio

**Affiliations:** 1 Faculty of Dentistry, University of Granada, Colegio Máximo de Cartuja s/n, 18071 Granada, Spain; 2 Medicina Clínica y Salud Pública Programme, University of Granada, 18071 Granada, Spain; 3 Laboratorio de Óptica, Instituto Universitario de Investigación en Óptica y Nanofísica, Universidad de Murcia, Campus de Espinardo (Ed. 34), 30100 Murcia, Spain

**Keywords:** collagen, membrane, bone regeneration, biomineralization

## Abstract

Natural extracellular matrix (ECM) collagen membranes are frequently used for bone regeneration procedures. Some disadvantages, such as rapid degradation and questionable mechanical properties, limit their clinical use. These membranes have a heterologous origin and may proceed from different tissues. Biomineralization is a process in which hydroxyapatite deposits mainly in collagen fibrils of the matrices. However, when this deposition occurs on the ECM, its mechanical properties are increased, facilitating bone regeneration. The objective of the present research is to ascertain if different membranes from distinct origins may undergo biomineralization. Nanomechanical properties, scanning electron (SEM) and multiphoton (MP) microscopy imaging were performed in three commercially available ECMs before and after immersion in simulated body fluid solution for 7 and 21 d. The matrices coming from porcine dermis increased their nanomechanical properties and they showed considerable mineralization after 21 d, as observed in structural changes detected through SEM and MP microscopy. It is hypothesized that the more abundant crosslinking and the presence of elastin fibers within this membrane explains the encountered favorable behavior.

## 1. Introduction

Guided bone regeneration (GBR) is a widely used clinical technique for peri-implant or periodontal bone augmentation. A barrier membrane is used to prevent non-osteogenic tissues from migrating into the bone defect, thereby permitting osteoprogenitors to grow at the defect area, exclusively [1,2].

The most employed barriers are collagen membranes [2]. They are mainly composed by type I collagen, which is a complex polymer. Collagen has a structure with four levels of hierarchy: (i) the primary level is amino acid triplets (mainly proline, hydroxyproline and glycyl); (ii) the secondary collagen structure is formed by these amino acids that stack repeatedly; (iii) the tertiary level is the triple helix composed by three interconnected α-chains; and finally (iv) a last hierarchy exists, forming collagen fibrils or fibers. Collagen fibrils continue to self-assemble linearly and/or laterally into networks, attaining different physicochemical properties at several tissues [3]. Two techniques are employed to obtain these collagen membranes: (i) the first involves the extraction, purification, and polymerization of collagen to form a functional biomaterial, and (ii) the other technique consists of decellularizing native tissues such as porcine or bovine pericardium, small intestinal submucosa, and dermis; these are called extracellular matrix membranes (ECM) [4]. Natural ECM membranes are porous and have a three-dimensional structure. They contain several preserved bioactive components, as growth factors, glycosaminoglycans, and glycoproteins [5]. It is possible that ECM membranes coming from different tissues may have distinct characteristics that should affect their function [2].

Collagen membranes are resorbable due to the instability of the collagen molecule. In brief, collagen is arranged into a triple helix structure, forming the collagen fibril and many fibrils are then arranged together with a covalent cross-linked bond to obtain a collagen fiber. The degree of cross-linking of collagen fibers will affect the rate of degradation with higher cross-linking producing slower degradation. However, during the membrane fabrication process the collagen crosslinking of natural tissues may be partially or completely lost [6,7].

The ideal barrier membrane does not exist. Collagen membranes possess certain disadvantages, such as insufficient mechanical properties and poor dimensional stability overtime. In an effort to address these problems, calcium phosphate (CaP) has been added to the membranes, as apatite crystallites are similar in chemical composition to the inorganic component of bone [8]. However, in order to ensure that collagen membranes have higher mechanical properties and stay longer in the clinical environment, a process of biomineralization is desirable. Through biomineralization, inorganic elements will be selectively deposited on specific organic macromolecules to produce biological minerals. It should be noted that collagen is frequently used as a universal template for biomineralization [9].

The objective of the present research was to ascertain differences in biomechanical properties, aging and biomineralization between commercially available collagen membranes from distinct origins.

## 2. Materials and Methods

### 2.1. Tested GBR Collagen Membranes Description

The analyzed membranes are CE-certified for surgery in oral applications. They are commercially available and have heterologous origins. The main membranes characteristics are summarized in the Table 1. The tested membranes were: (1) Derma (OsteoBiol^®^ by Tecnoss, Torino, Italy); (2) Evolution Standard (OsteoBiol^®^ by Tecnoss, Torino, Italy); and (3) Duo-Teck (OsteoBiol^®^ by Tecnoss, Torino, ^®^ by Tecnoss, Torino, Italy). According to the description made by the manufacturer, Derma is derived from porcine dermis and during the fabrication process the epithelial layer is removed. This membrane is composed of a network of highly purified non-crosslinked Type I and III porcine collagens fibers. The processing technique is performed at room temperature (cold process). These fibers are intermingled with porcine elastin fibers. Evolution Standard is a resorbable dense collagen mesh barrier derived from heterologous mesenchymal porcine pericardium tissue. Duo-Teck is derived from equine lyophilized collagen felt, one of the external surfaces is covered by equine bone particles (up to 300 µm).

### 2.2. Acellular Static In Vitro Bioactivity Test

Three specimens of each membrane were analyzed. Membranes were immersed in sterile flasks containing 20 mL of simulated body fluid solution (SBFS) [pH 7.45] for 7 and 21 days at 37 °C. Reagents per 1000 mL of SBF were: 8.035 g of NaCl, 0.355 g of NaHCO_3_, 0.225 g of KCl, 0.231 g of K_2_HPO_4_·3H_2_O, 0.311 g of MgCl_2_·6H_2_O, 39 g of 1 M HCl,0.292 g of CaCl_2_, 0.072 g of Na_2_SO_4_, 118 g of Tris, 0–5 mL of 1 M HCl for final pH adjustment. All the experimental conditions were as specified at the ISO standard 23317:2014 [10].

### 2.3. Nanomechanical Properties Analysis

Nanomechanical properties were determined using the Hysitron Ti Premier nanoindenter together with a commercial nano-DMA package (Hysitron, Inc., Minneapolis, MN). A fused quartz sample was used to calibrate the nanoindenter tip. In order to maintain contact between the tip and the sample surface a quasistatic force setpoint of 2 μN was employed. A dynamic and oscillatory force of 2 μN was superimposed on the quasistatic signal at a frequency of 100 Hz. Based on a calibration-reduced modulus value of 69.6 GPa for the fused quartz, the best-fit spherical radius approximation for tip was found to be 85 nm for the selected nano-DMA scanning parameters. To acquire the modulus mapping of our samples a quasistatic force setpoint (Fq = 2 μN) was used to which a sinusoidal force of frequency f = 100 Hz and amplitude FA = 0.10 μN was superimposed. The resulting displacement (deformation) at the site of indentation was monitored as a function of time. To maintain the hydration of the samples while eliminating problems related to the meniscus forces transferred from droplets of fluid to the indenter [11], a drop (1.5 mL) of 99.4% ethyleneglycol was applied to the sample surface [12].

Three randomized regions 5 × 5 μm in size were scanned at each surface using a frequency of 0.2 Hz. Ten complex modulus, loss modulus, storage modulus and Tan δ data were collected from each of these scanned regions. Under steady conditions (application of a quasistatic force), the indentation modulus of the tested sample, E, can be obtained by application of different models that relate the indentation force, F, and depth, D [13].

### 2.4. Scanning Electron Microscopy (SEM)

After nano-DMA analysis, specimens were cut in two halves. One half was fixed in a solution of 2.5% glutaraldehyde in 0.1 mol/L sodium cacodylate buffer for 24 h. Samples were subjected to critical point drying (Leica EM CPD 300, Wien, Austria), sputter-coated with carbon by means of a Nanotech Polaron-SEMPREP2 sputter-coating equipment (Polaron Equipment Ltd., Watford, UK). Samples were then observed with a field emission scanning electron microscope (FESEM Gemini, Carl Zeiss, Oberkochen, Germany). Employed accelerating voltage was 3 kV, and 3.0–3.6 mm was used for working distance. Images were taken with different magnifications at randomized areas, in each specimen. Elemental analysis was done by means of an energy dispersive analysis system (EDX) (Inca 300 and 350, Oxford Instruments, Oxford, UK).

### 2.5. Multiphoton Microscopy Imaging

The second half of each specimen was submitted to multiphoton (MP) microscopy analysis. Image acquisition was performed by means of a custom MP microscope. Two different MP signals were obtained herein: second harmonic generation (SHG) from the collagen-based components and two-photon excitation fluorescence (TPEF) or autofluorescence from different proteins present in the samples. In brief, a femtosecond Ti:Sapphire laser (800 nm) was used as illumination source. The beam passed through all optical elements and was focused by a dry long-walking distance objective on the sample’s plane. The emitted MP signal was collected through the same objective and was detected by the detection unit composed of a photomultiplier tube and a photon counter. Two spectral filters placed in front of this detection unit were used to isolated the SHG signals (narrow-band spectral filter, 400 ± 10 nm) and the TPEF (broadband spectral filter, 435–700 nm) signals [14,15].

For each sample involved in this study, pairs of images SHG/TPEF were acquired. Within each sample, three different and randomly chosen areas were imaged. For each image a number of parameters were calculated: total intensity (i.e., MP signal) and roughness. In addition, an additional parameter combining both signals was also computed. This parameter represents the contrast of both MP signals and is often known as the Aging Index [16]. It has been employed to study the aging changes suffered by biological collagen-based tissues.

$$Age\;Index=\frac{I_{SHG}-I_{TPEF}}{I_{SHG}+I_{TPEF}}$$

### 2.6. Statistical Analysis

Data normal distribution was assessed by Kolmogorov–Smirnov (*p* > 0.05). ANOVA and Student–Newman–Keuls multiple comparisons tests were performed to compare between experimental groups (*p* < 0.05). The IBM SPSS Statistics 24 computer software was employed.

## 3. Results

### 3.1. Nanomechanical Properties Analysis

Mean and standard deviation of complex modulus, storage modulus and tan delta attained for the different membranes, at the different immersion points, are presented in Table 2.

**Figure 1:**
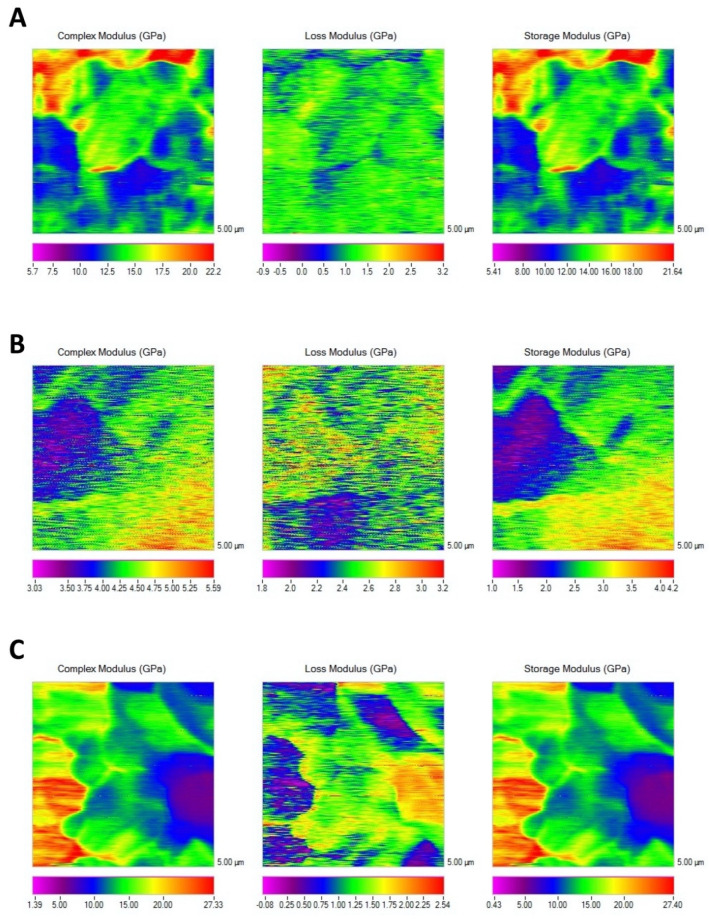
Nano-DMA mapping of the three membranes at the initial time-point. (**A**) Derma, (**B**) Evolution and (**C**) Duo-Teck. Scale bars are in GPa. Scan size is 5 μm × 5 μm.

**Figure 2:**
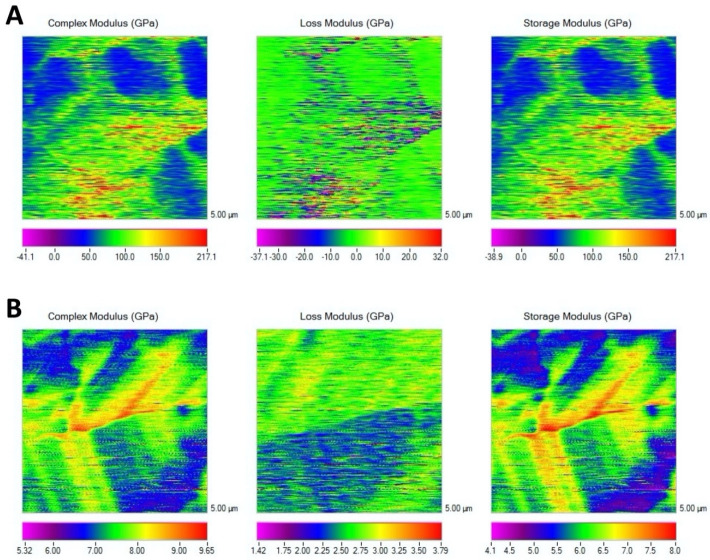
Nano-DMA mapping of the three membranes after 21 d of SBFS immersion. (**A**) Derma and (**B**) Evolution. Scale bars are in GPa. Scan size is 5 μm × 5 μm.

The complex modulus mapping of membranes surfaces is presented in Figure 1 and Figure 2. Before immersion, Evolution was the membrane attaining the lowest mechanical properties (*p* < 0.05), and no difference was found between Derma and Duo-Teck. Duo-Teck membrane did not resist 1 week of immersion; therefore, mechanical values were not determined for any immersion time period. After 7 days of storage, the mechanical properties of Derma membranes increased (double-fold for Derma), but no differences were found between both membranes (*p* = 0.1). After 21 days, Derma once again increased mechanical modulus (almost twofold) (*p* < 0.05). Evolution did not change in mechanical properties, over time (*p* > 0.05). A similar trend was encountered for storage modulus.

### 3.2. Scanning Electron Microscopy (SEM)

**Figure 3:**
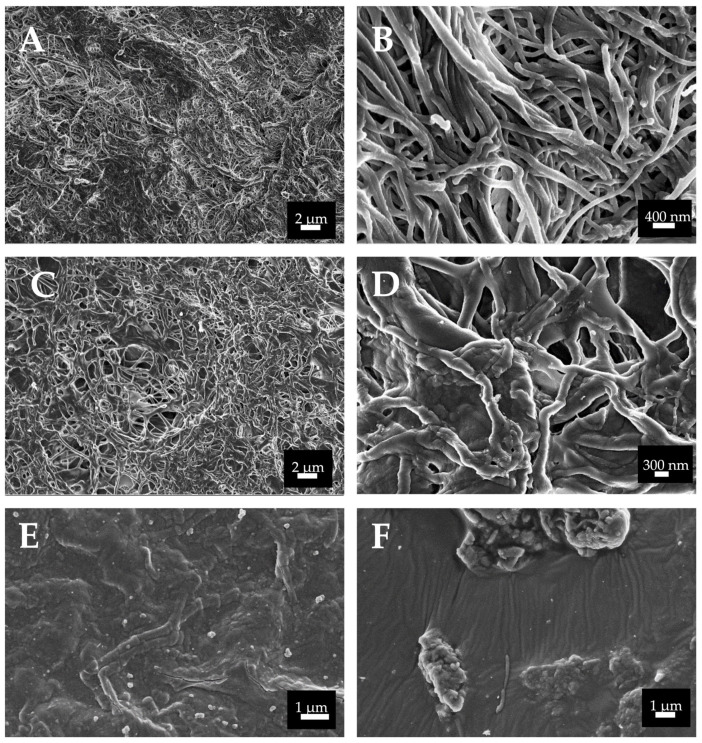
SEM images of the three membranes at the initial time-point. (**A**,**B**) Derma; (**C**,**D**) Evolution; (**E**,**F**) Duo-Teck. Randomly distributed collagen fibrils are found in Derma and Evolution membranes. Higher magnification of fibril bundles reveals the typical periodic banding pattern of collagen fibrils. Images have different magnifications (6000× to 40,000×).

The SEM images are presented in Figure 3. There are clear differences between the microstructures of the tested membranes. Derma (Figure 3A,B) and Evolution (Figure 3C,D) showed a hierarchical 3D interconnected porous structure with a rough surface. At Derma, the collagen fibers were densely packed and formed multidirectional bundles; and only small pores are observed (Figure 3A,B). Evolution and Derma are composed by collagen fibers of about 0.5 micron in diameter (Figure 3). Collagen matrix at Evolution seems to be less dense than Derma. At Derma surfaces, some other fibers are evidenced and distinguished from collagen, due to their higher size (about 1 micron in diameter) (Figure 3A,B). In some specific zones, characteristic collagen fibers striation showing crosslinking was observed at Evolution (Figure 3D) and Derma membranes (Figure 3B). Crosslinked fibers were much more abundant in Derma than in Evolution membranes (Figure 3B,D). Duo-Teck presents a smooth surface, and it appeared as a corrugated layer with somewhat oriented collagen fibers. At Duo-Teck surfaces some agglomerates of particles are randomly distributed (Figure 3E,F).

**Figure 4:**
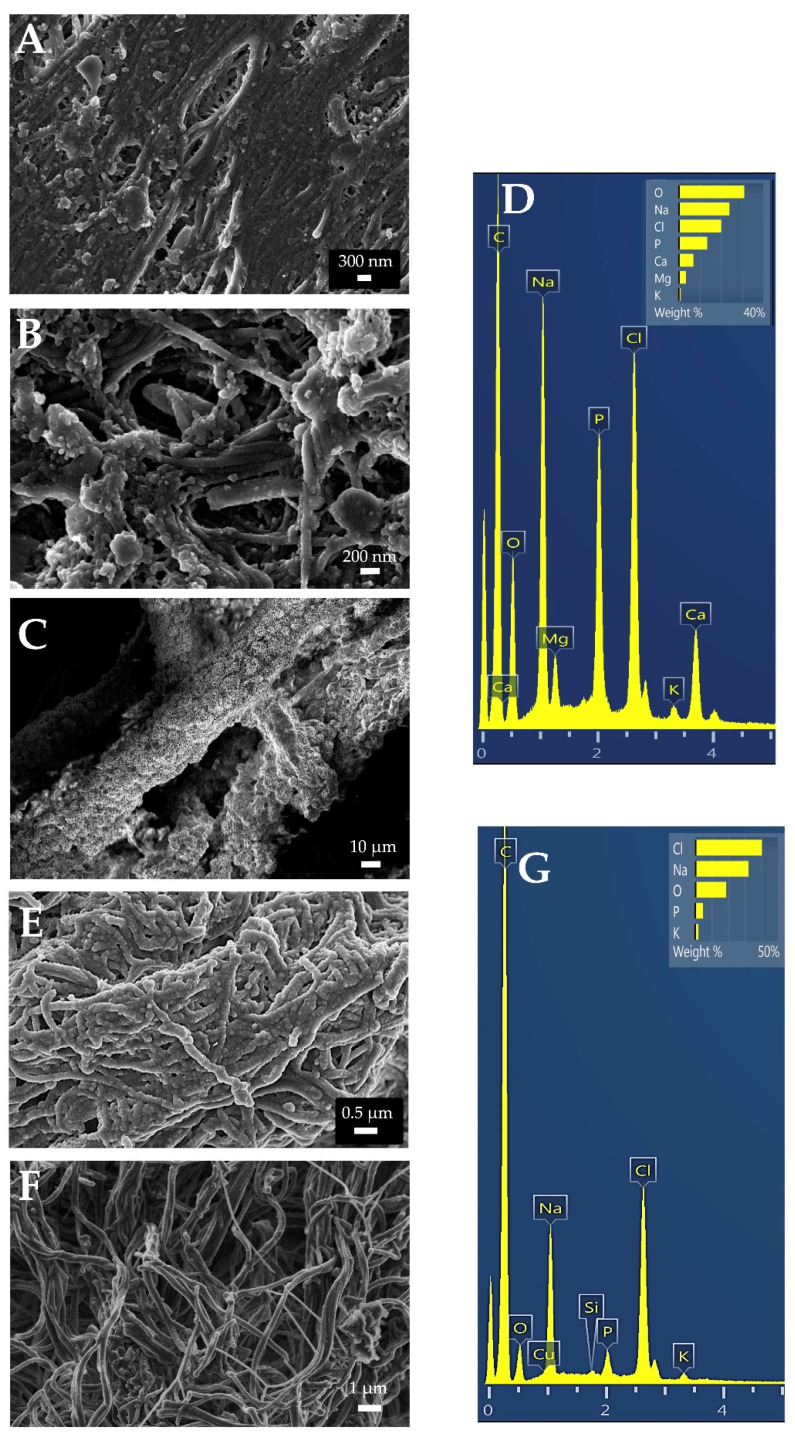
SEM images taken from the three membranes after 21 d of SBFS immersion. (**A**–**C**) Derma, (**D**) EDS spectrum from Derma membrane showing calcium and phosphate presence. (**E**,**F**) Evolution, (**G**) EDS spectrum from Evolution membrane. Randomly distributed collagen fibrils are found in all the membrane types. Some zones evidencing rounded deposits for both membranes are shown. At EDS analysis, elements such as C or O can be detected for their presence, but cannot be quantified reliably. Images have different magnifications (1000× to 50,000×).

The membranes’ microstructure was only slightly changed after 21 d of immersion. In the Derma specimens, a superficial mineral formation layer is evidenced on several of the analyzed areas (Figure 4A). At higher magnification (Figure 4B), some crosslinked collagen fibers and new deposits may be observed. In some selected zones, at the periphery of the samples, mineralized fibers of more than 30 µm in diameter were encountered (Figure 4C). After EDS, calcium and phosphate were detected (Figure 4D). At Evolution membranes, separation of collagen fibers occurred during these 21 days. The outer layer had degraded enough in order to expose the underlying interconnected collagen pores (Figure 4E). Collagen fibers are thicker and have a rough appearance (Figure 4F). At the background twisted, and bent collagen bundles are present. After EDS, calcium and phosphate were not detected at Evolution membranes (Figure 4G).

### 3.3. Multiphoton Microscopy Imaging

**Figure 5:**
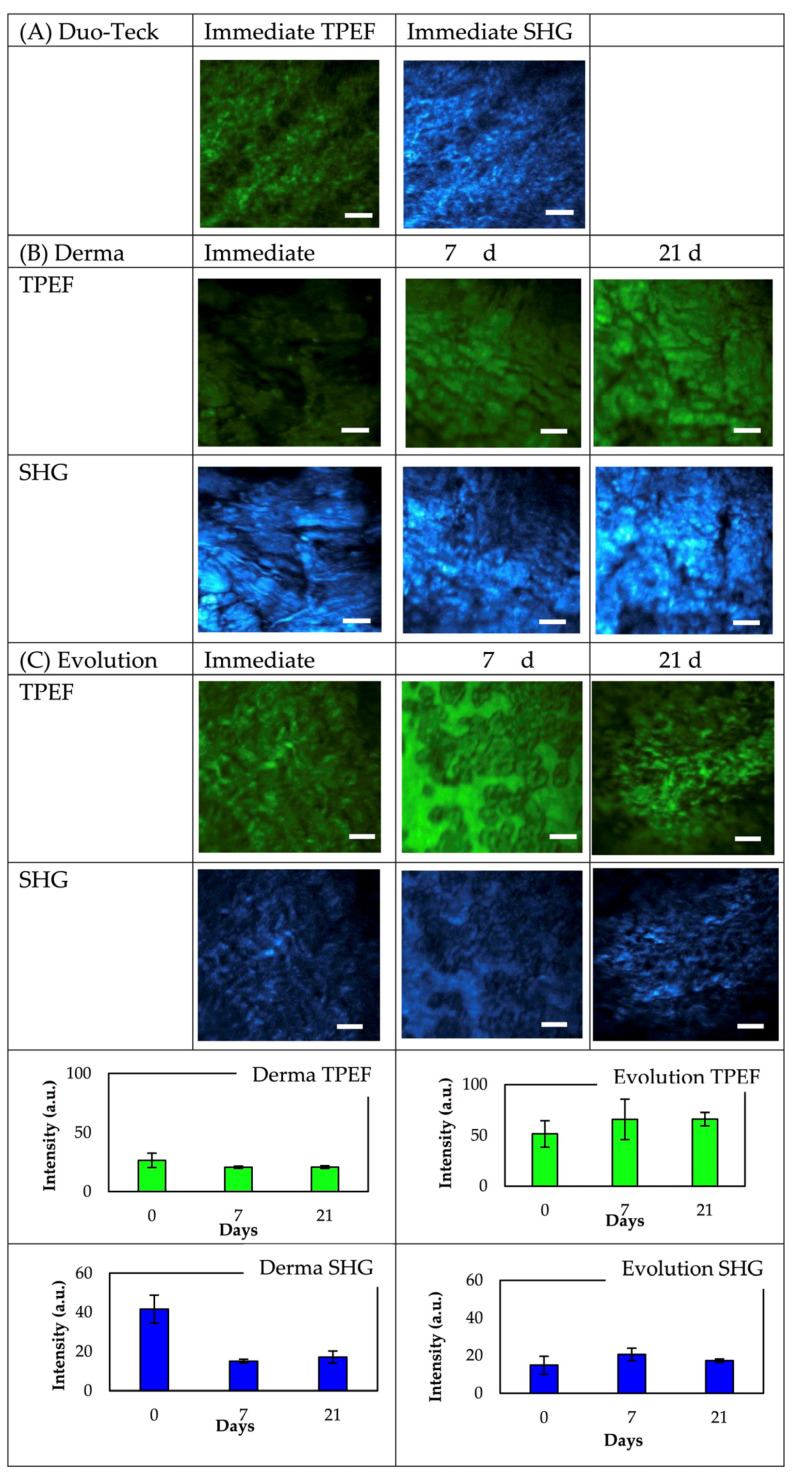
TPEF and SHG images of representative membranes. Scale bar: 25 μm.

The two different collagen membranes were examined initially, after 7 d, and after 21 d of immersion in SBFS by using MP microscopy. In the collagen membranes, both the SHG emission of collagen fibers and the (autofluorescence) TPEF signal from other different proteins were monitored; therefore, structural alterations were ascertained with label-free imaging. The results of TPEF and SHG analysis are shown in Figure 5.

The bottom plots in Figure 5 show that at 800-nm excitation wavelength and independently of the assessment time-point, a more intense TPEF signal could be detected in the Evolution samples. The TPEF signal intensity was approximately 50% higher than in the Derma membranes group. TPEF signal remained fairly constant in both groups. None of the two sets of samples showed significant changes in the TPEF signal over time.

On the contrary, the SHG signal intensity was higher in Derma samples (about two-fold) before immersion in SBFS and both membranes showed a different behavior over immersion time: the SHG signal diminished in Derma and no changes were detected in Evolution.

**Figure 6:**
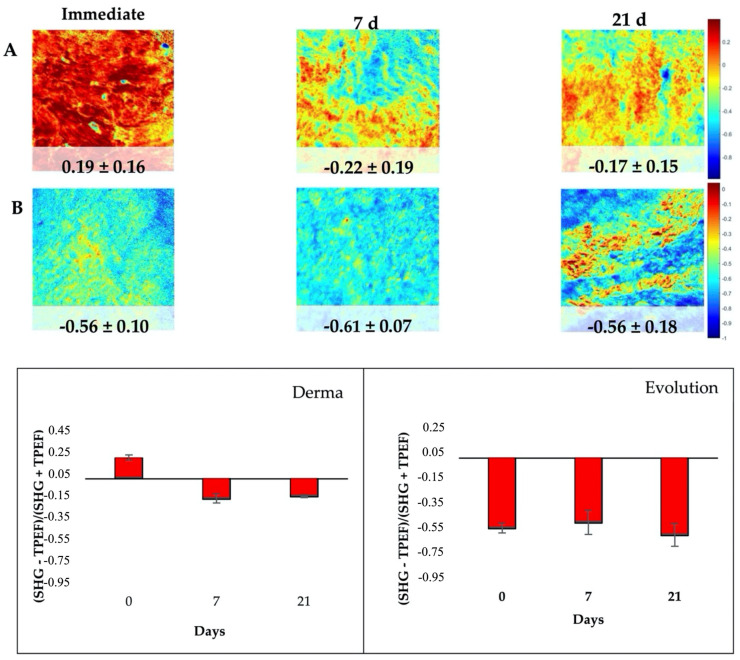
Aging Index map of Derma ((**A**), upper panels) and Evolution ((**B**), middle panels) at immediate (left column), 7 d (middle column) and 21 d (right column)of immersion. Plots show the mean and the standard deviation values of the index across the maps for all imaged areas for Derma (left) and Evolution (right) at the different immersion time-points.

The changes after immersion were also quantified by means of the Aging Index parameter. The two membranes displayed a different behavior. The spatially resolved map for both membranes at each immersion time was presented in Figure 6. As expected (see plots in Figure 5), no changes were found in Evolution. In contrast, a change in the sign of the Aging Index was found in Derma, what is associated to a decrease in SHG signal (respect to TPEF) with immersion time. The plots depict the corresponding mean and the standard deviation values across the maps for every sample and time point.

## 4. Discussion

Collagen membranes should be biocompatible, porous, biodegradable, and ideally osteoconductive and osteoinductive to facilitate bone regeneration [1]. Different commercially available collagen membranes have distinct estimated degradation times, which are sometimes shorter than the desired in order to produce predictable clinical outcomes in bone regeneration procedures [3].

Collagen membranes biomineralization may lengthen degradation times. In biomaterial science, bioactivity denotes that the material is able to form calcium phosphate mineral on its surface, after in vitro immersion in SBFS [10,17]. This property may rely on the material surface chemistry and microstructure, which should allow mineralization to occur. Collagen fibrils are frequently used as mineralization templates, as there are nanoscopical channels present within these fibrillar structures and they enable ordered deposition of minerals [9]. The understanding of biomineralization mechanisms is crucial for promoting intrafibrillar mineralization and for developing more durable bioinspired materials. The biomineralization of type I collagen is mainly characterized by intrafibrillar mineralization, in which hydroxyapatite (HAp) is deposited within the gap zone of collagen fibrils; extrafibrillar mineralization does also occur, when HAp deposits are on the surface of collagen fibrils. However, the mechanisms driving these two processes remain unclear and are somehow debatable [3,18]. Achievement of intrafibrillar mineralization of non-crosslinked collagen fibers cannot be obtained only by immersing collagen matrices in mineral ions supersaturated solution. As has been demonstrated it requires the use of nucleation inhibitors that will stabilize the ion association complexes, in order to prevent them from crystallizing outside of the collagen fibrils [9].

The employed heterologous collagen membranes have undergone chemical and physical processes of decellularization and sterilization in order to overcome immunogenic responses or infections at the clinical environment [19]. It has been previously reported that when these processes are performed in pericardia, collagen fibers are denaturalized, glycosaminoglycans content is reduced, and the natural collagen cross-linking is altered. These changes make membranes more susceptible to degradation and less prone to biomineralization, being also detrimental on their biomechanical behavior [20].

SEM was performed to assess the superficial collagen structure of the porcine membranes. At Derma and Evolution, collagen fibrils are randomly distributed forming undulating collagen bundles that are loosely arranged. As the packing of fibrils into fibers was not very tight, it results in single fibrils or small bundles which may interconnect larger bundles. In the case of Evolution, a loose meshwork was formed by isolated fibrils running crosswise (Figure 3). For Derma, bundles of different sizes were aligned generally, in parallel and irregular spaces were separating from each other. The collagen fibrils sometimes were intertwined with each other. For a given membrane, the fibrils were very homogeneous and uniform in shape. However, the geometric organization of collagen fibers in dermis and its relationship to that of elastic fibers still remain unclear. It has been speculated that the tight packing and complex intertwining of dermal collagen fibers may hinder accurate analysis of fiber orientation. There are also optical and scanning electron microscopy studies suggesting that the network of collagen fibers is basically random [21,22].

Microfibrils are subunits of the collagen fibril. It depends on tissue if the collagen fibrils exhibit a diameter from 10 to more than 500 nm. At the analyzed membranes, collagen fibers possess a diameter of about 1 micron (Figure 3). It is not easy to estimate the length of the collagen fibrils in the different tissues, as fibril ends are rarely observed at micrographs. Collagen fibrils may reach a length of several mm and are randomly distributed (Figure 3) [23]. In some parts of the scanning electron microscopy image, collagen fibers were found to show the periodical striations, being a proof of crosslinking. The collagen bands show an average D-periodicity of about 70 nm (Figure 3B). These membranes are not treated to acquire cross-linking during the fabrication process. However, we should consider that cross-linking happens physiologically in native tissue; therefore, different animal tissues can provide collagen with distinct crosslinking degrees. The persistence of crosslinking after the membranes’ commercial preparation might produce an increase in collagen mechanical properties and durability, being an alternative to artificial chemical cross-linking, which has been found to evocate some adverse effects [22]. However, only limited knowledge exists about this topic.

Duo-Teck is collagen in a lyophilized state. Fibers are not distinguishable (Figure 3E,F). Duo-Teck membranes possess micro-sized bone particles of around 10 to 30 microns (Figure 3E,F). These particles are probably responsible for the high mechanical properties at the initial state (Table 2, Figure 1). However, they seem to be dissolved after 7 d of SBFS immersion. It is a membrane highly susceptible to biodegradation [22]. It was not possible to perform any mechanical or multiphoton microscopy measurements, after 7 d. It may be that the re-hydration and lyophilization procedures during membrane manufacturing led to the loosening of connections between collagen fibers, making them highly susceptible to the hydrolytic processes [24]. At Duo-Teck membranes, mineralization is not observed, even when bone particles are initially present.

Mechanical properties of collagen membranes are important not only for clinical manipulation but also to support for intraoral forces during bone regeneration [1]. At the initial state, Evolution from porcine pericardium seems to have the lowest mechanical behavior. In general, Derma from porcine dermis attained the highest mechanical performance (Table 2). After SBFS immersion, overtime increases in mechanical properties do also indicate the existence of fiber mineralization, and the lowering may be produced by collagen denaturation [8]. When Derma is compared to Evolution, a distinct mechanical behavior is encountered, even when both are porcine-derived membranes, confirming that the tissue of origin is also an important parameter to predict membrane properties and durability [22,25,26,27].

As previously mentioned, Evolution is a heterologous porcine membrane obtained from pericardium. It is mainly composed by collagen fibers and a few elastin fibers that are not homogeneously distributed. Elastin presence seem to be scarce at deep layers [28]. Even when some rounded deposits were found at some zones of Evolution membranes (Figure 4E), calcium and phosphate presence was not detected by EDS (Figure 4G). Therefore, no biomineralization was produced after 21 d of SBFS immersion. Increases in nanomechanical properties were neither evidenced. This is in accordance with some previous reports in which collagen membranes from porcine pericardium were shown to be non-mineralizable, or less susceptible to mineralization than bovine pericardium or other collagen membranes. Therefore, they are recommended for cardiovascular clinical applications [29].

Even when Derma is also a heterologous ECM membrane from porcine origin, a different result is obtained after SBFS immersion, which is able to produce some kind of biomineralization (Figure 4A–C). These results are in accordance with the observed increase in nanomechanical properties (Table 2, Figure 1 and Figure 2), being significantly different from Evolution after 21 d of SBFS immersion. One of the most important differences in these membranes’ composition is the presence of abundant elastin fibers which will increase their mechanical properties. Elastin may confer to Derma high mechanical properties and moreover, the elastic behavior of elastin is related with the plasticizing effect of water. The hydration state of elastin includes extrafibrillar and intrafibrillar water, and the latter one is tightly bound to the polypeptide chains. As has been stated in previous investigations, there is another possible mechanism of the plasticizing effect of water on elastin; at nanometric scale it may be attributed to the replacement of protein/protein hydrogen bonds by protein/water hydrogen bonds, as the latter will increase the chain mobility. There may also exist realignment of elastic fibers. Decrease in spacing induced by water loss may likely lead to an increase in the density of elastic fibers and potential fiber realignment. The much denser fiber network structure will probably result in a stiffer mechanical behavior [30]. Elastic fibers endow the tissue with elasticity and resilience, thus allowing repeated stretch and the subsequent passive recoil [23]. Therefore, the high attained mechanical properties are probably due to elastin, which is responsible for elasticity and resilience in many tissues, and is well-known for its extreme durability and ability to deform reversibly. High mechanical properties of cross-linked elastin fibers have been previously described and therefore, these membranes from porcine dermis have been previously recommended for abdominal surgery [31]. Derma has also been reported to be highly resistant to degradation [22], and this condition may also help in supporting the formation of mineral deposits on its surface, previously hypothesized by Toledano et al. [22]. Elastin is an insoluble polymer formed by cross-linking of the monomeric soluble precursor, tropoelastin [32]. It should be taken into consideration that elastogenesis has an almost absent turnover, elastin being a highly stable molecule. The extensive and durable crosslinking of elastin is important not only for elastic properties but also for considering the insolubility of these membranes. Therefore, elastin is highly resistant to degradation by acidic and alkaline chemical agents and by many proteases [32]. The calcium-binding capacity of elastin due to the presence of mineralization nucleation sites as sulfhydryl and carboxyl groups has also been described [33]. An additional cause of major mineralization may also be the higher abundance of collagen crosslinking encountered in Derma if compared to Evolution (Figure 3), since collagen crosslinking favors biomineralization [9].

It may also be observed at the different images, that mineral deposition and hydroxyapatite crystallization occur in different manners (Figure 4B,C). This fact may happen because intermediate amorphous or poorly crystalline phases are evolving from amorphous (Figure 4B) to more crystalline apatite phases (Figure 4C). The mechanism underlying this mineralization process has been previously found to be the existence of negatively charged carboxylate groups generated by cleavage of peptide bonds in the fragmented elastin or collagen fibrils which can interact with calcium ions of SBF media [33,34].

Present results may also be related to the hydrophilicity process and to the potential in vitro degradability of these membranes. Significantly major degradation resistance has been previously found for Derma when compared to Evolution and Duo-Teck, if immersed at phosphate buffered and at other different enzymatic solutions [22]. This greater stability is consistent with the presence of elastin fibers in Derma membranes, and with the higher crosslinking of collagen evidenced for Derma, in the present research. The highest degradability was reported for Duo-Teck, from equine lyophilized collagen felt. Sings of complete degradation started to appear as soon as after 8 h of immersion in the different solutions [22]. Findings by Fickl et al. [35], who reported a slow resorption of Derma membrane in beagle dogs after four months, are also in accordance with this. The membranes almost preserved the original thickness after four months. These new observations can explain the major durability of the ECM membrane from porcine dermis. It will also facilitate strategies to control the membranes’ degradation processes and their mechanical properties, by promoting the early calcium phosphate deposits formation. It will lead to the enhancement of the tissue engineering applications of these three-dimensional matrix scaffolds.

The attained high mechanical properties are also important for cells. It has been suggested that substrate stiffness (tan delta) and matrix elasticity (complex modulus) may be probed by cells [36], which may modify proliferation and even differentiation as a response to the encountered differences in mechanics of fibrillar matrices [37]. Therefore, membranes with high nanomechanical properties may enhance cell adhesion, osteoblasts differentiation and proliferation [38]. Tan delta values greater than 1, represent liquid-like regions and tan *δ* values lower than 1 represent gel-like behavior [39]. In this regard, Derma and Evolution membranes may potentially favor osteoblasts cell differentiation and spreading since their tan *δ* values ranged from 0 to 0.5 and from 0.4 to 0.6, respectively (Table 2).

MP is an emerging imaging method. It is based on non-linear optical effects produced by near-infrared femtosecond lasers. Two MP imaging modes are broadly used: SHG and TPEF [21]. TPEF signal arises from flavins and other intrinsic chromophores such as NAD(P)H and elastin. SHG allows the visualization of non-centro symmetric structures such as type-I fibrillar collagen. However, it has also been shown that SHG microscopy is able to reveal structural changes produced on tissues [40]. Therefore, it is a powerful tool that may help to characterize the architecture of collagen fibers. Collagen fibers are able to provide SHG signal due to their structural regularity. Hence, MP can reveal the structural order and rearrangement [41,42,43]. However, when using near-infrared light, a laser beam can penetrate deeper; nevertheless, it is not possible to visualize collagen fibers at depths of ≥100 μm, mainly due to light scattering [40]. It is a limitation of this study which may also affect the attained results.

When the collagen fibers lose their structural regularity, the emitted SHG signal is reduced [44]. Monitoring the SHG signal through time may provide an indication of changes in collagen’s structural arrangement. When analyzing Derma specimens, two-photon microscopy showed that second harmonic-generated (SHG) signal intensities significantly decreased with time of immersion, and no changes were detected when the autofluorescence signal was observed (Figure 5). At Derma membrane, SHG and TPEF signals are evenly mixed. In Evolution, proportions of TPEF and SHG signals do not vary with immersion time after 7 and 21 days (Figure 5). After SBFS immersion, the degradation of proteins may occur, thus leading to the lowered SHG signal intensity in collagen membranes [41]. However, in the present model it is observed that Evolution did not change the MP signals overtime; therefore, it is hypothesized that the attained changes in SHG signals in Derma membrane (Figure 5) may be caused by the biomineralization process that implies structural modification of collagen fibers. As in Derma specimens, the orientation of collagen fibers did not change during the SBFS immersion periods, but the fibers stayed parallel forming randomized bundles. Thus, the accumulation of minerals on their surfaces may have led to the lower emitted SHG signal, without inducing major structural degradation. This observation was also confirmed by SEM analysis. However, understanding the exact mechanisms behind this finding awaits further exploration.

The aging process of collagen membranes was also investigated by the use of TPEF+SHG microscopy. The changes in aging are quantified by a SHG to the autofluorescence aging index of collagen membranes [16]. Once more, the two membranes displayed a distinct behavior. No changes were found in Evolution but in Derma, areas of SHG relative to TPEF decreased with aging (immersion time) (Figure 6). As the TPEF and SHG images of the superficial collagen membranes were acquired, the results were consistent with the mechanical and histological findings in which collagen SHG signals seems to diminish progressively, and no changes or mineralization occurred on elastin fibers. A similar trend of the changes in the proportion of elastic tissue and collagen fibers is hypothesized through analysis of the histological findings.

Other different synthetic polymers are promising alternatives to conventional collagen membranes in the effort to accomplish greater mechanical stability and longer degradation times. Synthetic electrospun polymeric nanofibers made of polycaprolactone, poly-l-lactic acid, or other different polymers have been proposed because of their cellular recognition properties, major resistance to degradation, and biocompatibility [43,44,45]. In some cases, to increase the biomimeticity of these scaffolds toward bone tissue, these nanofibers have also been mineralized [43]. This is currently a focus of research on polymeric biomaterials science.

To the best of our knowledge, this is the first time that a nano-scaled analysis of commercially available collagen membranes for GBR has been performed. Such analysis is crucial, since the membrane properties, including the diameter, spacing, and orientation of fibers, as well as membrane stiffness, have been shown to modulate cellular behaviors such as proliferation, migration, and differentiation [23]. Since differences in collagen tissue structure led to different biological responses, their specific potential in the different areas of regenerative medicine should be taken into account.

## 5. Conclusions

It may be concluded that the origin of the collagen membrane influences its physicochemical behavior. The presence of elastin fibers in collagen membranes seems to favor mechanical properties and/or biomineralization, therefore resistance to degradation may also increase in membranes containing elastin fibers.

Two significant trends in modern GBR procedures may be facilitating membranes biomineralization (using naturally derived materials and/or employing principles of tissue engineering) and searching for additional and more appropriate native tissue sources of GBR collagen membranes.

Finally, the use of nonlinear optical imaging might be encouraged for in situ high-resolution collagen-derived biomaterials structure determination, as a supplemental procedure.

## Figures and Tables

**Table 1:** Commercial name, collagen type and origin of the membranes. The information was obtained from the membrane providers.

Commercial Name	Collagen Type	Origin	Cross-Link	Others
Derma	Type I and III	Heterologous porcine dermis	No	Elastin fibers
Evolution Standard	Collagen fibers	Heterologous porcine pericardium	No	
Duo-Teck	Liophilized felt	Equine	No	Bone particles

**Table 2:** Nanomechanical properties measured on the different membranes after the different periods of immersion (expressed as mean and standard deviations -SD-). Distinct letters indicate statistically significant differences between membranes and numbers between immersion periods (*p* < 0.05).

		0 d		7 d		21 d		
		Mean	SD	Mean	SD	Mean	SD	Statistics
**Complex modulus**	Derma	14.02 A1	3.51	40.03 A2	18.78	71.60 A3	37.00	F = 43.15; *p* < 0.0001
	Evolution	4.47 B1	0.68	9.97 B1	13.10	8.34 B1	1.07	F = 133.73; *p* < 0.0001
	Duo-Teck	13.56 A	3.68	-	-	-	-	
Statistics		F = 98.99; *p* < 0.0001		t = 1.45; *p* = 0.15		t = 9.36; *p* < 0.0001		
**Loss modulus**	Derma	1.31 A1	0.49	0.97 A1	2.84	−0.77 A1	11.81	F = 0.75; *p* = 0.47
	Evolution	2.60 B1	0.43	0.51 A2	1.79	2.61 B1	0.36	F = 37.40; *p* < 0.0001
	Duo-Teck	1.43 A	0.58	-	-	-	-	
Statistics		F = 60.78; *p* < 0.0001		t = 0.75; *p* = 0.46		t = 1.57; *p* = 0.12		
**Storage modulus**	Derma	13.19 A1	3.49	39.56 A2	17.72	73.92 A3	36.84	F = 49.58; *p* < 0.0001
	Evolution	2.82 B1	0.92	6.77 B1	12.88	7.02 B1	1.06	F = 171.32; *p* < 0.0001
	Duo-Teck	13.43 A	3.42	-	-	-	-	
Statistics		F = 134.05; *p* < 0.0001		t = 0.97; *p* = 0.33		t = 9.94; *p* < 0.0001		
**Tan delta**	Derma	0.19 A1	0.05	0.09 A1	0.55	0.09 A1	0.21	F= 0.79; *p* = 0.45
	Evolution	1.02 B1	0.35	0.13 A2	0.40	0.52 B3	0.10	F= 60.68; *p* < 0.0001
	Duo-Teck	0.24 A	0.19	-	-	-	-	
Statistics		F = 120.22; *p* < 0.0001		t = 0.35; *p* = 0.72		t = 10.04; *p* < 0.0001		

## Data Availability

The data presented in this study are available on request from the corresponding author.
